# ERP correlates of word onset priming in infants and young children

**DOI:** 10.1016/j.dcn.2013.12.004

**Published:** 2014-01-08

**Authors:** Angelika B.C. Becker, Ulrike Schild, Claudia K. Friedrich

**Affiliations:** a Independent Research Group Neurodevelopment, Department of Psychology, University of Hamburg, Hamburg, Germany; b Developmental Psychology, Department of Psychology, University of Tübingen, Tübingen, Germany

**Keywords:** Language acquisition, Infants, ERPs, Phonological priming, Word onset priming, Lexical–phonological processing

## Abstract

Using word onset priming with early learned words, we tracked access to phonological representations and predictive phonological processing at 6, 12, 18, and 24 months after birth. Event-Related Potentials (ERPs) were recorded while participants heard German word onsets (primes) followed by disyllabic spoken words (targets). Primes and target onsets were either congruent or incongruent (*ma* – *Mama* vs. *so* – *Mama* [Engl. ‘mommy’]). For an adult control group, ERP differences were found for the N100 complex, which has been related to abstract auditory analysis; and for the P350 deflection, which has been related to lexical access. A combined analysis of all infants and young children revealed an immature instance of an N100 effect, suggesting adult-like abstract speech sound processing. A central negativity effect, which had formerly been obtained when adults or older children were engaged in a lexical decision task, suggests that adult-like predictive phonological processing is available early in infancy. However, the absence of a P350-like effect in the infant data suggests that adult-like access to phonological forms is not established in the first two years of life. Taken together, ERPs recorded in word onset priming proved useful in investigating early phonological processing without an explicit behavioral measure.

## Highlights

• Word onset priming appears to be a powerful method for investigating early lexico-phonological processes.
• Based on word onsets, infants seem to make phonological predictions about word forms.
• Adult-like word form access seems to develop only after the second year of life.

## 1 Introduction

From early on, infants store phonological representations of words that frequently occur in their caregivers’ speech. For example, 4.5-month-olds prefer to listen to their own name (Mandel et al., 1995). Six-month-olds prefer to look to video images of their mommy or daddy when hearing *mommy* or *daddy* respectively (Tincoff and Jusczyk, 1999); they prefer to look to at videos of an adult's hand or feet when hearing *hand* or *feet* respectively (Tincoff and Jusczyk, 2012); and they prefer to look to concrete familiar objects like *banana* when hearing the respective label (Bergelson and Swingley, 2012). Whether the format of early phonological representations and their access mechanisms are compatible for infants, young children and adults is still a matter of debate. Several aspects that characterize adult spoken word recognition have been in focus of behavioral research with young children, namely (i) incremental activation of word form representations as the speech signal unfolds in time (Marslen-Wilson, 1987); (ii) graded activation of word form representations as a function of their goodness-of-fit with the speech signal (see Dahan and Magnuson, 2006, McQueen, 2007); and (iii) competition between activated entries (see Luce, 1986, McClelland and Elman, 1986).

Behavioral results obtained with the preferential looking paradigm have been interpreted as evidence for incremental gradual activation in young children. When 18-month-olds hear the onset syllable of a word like *baby (/bei-/)* and are presented with a picture of a *dog* and a *baby*, they fixate longer on the target picture with an overlapping onset than on the unrelated picture (Fernald et al., 2001). That is, comparable to adults, word recognition in 18-month-olds does not appear to wait until the complete word is presented. Furthermore, 14- and 15-month-olds (Swingley and Aslin, 2002) as well as 18- and 24-month-olds (Swingley and Aslin, 2000) tolerate one-phoneme mispronunciation in preferential looking. For example, when hearing *tog* the proportion of children's fixations of the picture of a *dog* is enhanced compared with fixations of an unrelated distractor. However, compared with the correct pronunciation, children fixate less on the target picture when they listen to the mispronunciation. This attests that already very young children pay attention to segmental variation in phonological word forms.

However, results obtained with the “Switch task” have suggested that adult-like gradual activation as indexed by the adaptive capacity to use mispronunciation is not robust during the first two years of life (e.g., Stager and Werker, 1997, Werker et al., 2002). Here, fixation latencies were used to test whether children who had been familiarized with novel objects and respective new labels, such as *bih*, tolerate slight mispronunciation of the new labels, such as *dih*. A *u*-shaped developmental pattern was found: fixation latencies in 8-month-olds (Stager and Werker, 1997), 18-month-olds and 24-month-olds (Werker et al., 2002) dishabituate to the mispronunciation, reflecting that those infants and young children are able to distinguish between the two labels in this task. However, 14-month-olds failed to dishabituate (Stager and Werker, 1997), suggesting that the first word form representations are not detailed. Event-Related Potentials (ERPs) corroborate this pattern of behavioral results: ERPs of 20-month-olds, but not of 14-month-olds differentiated between words that were known to the young children (e.g., *bear*) and pseudowords that slightly varied from the words (e.g., *gare*, Mills et al., 2004).

Evidence for competition between activated word candidates is restricted to the end of the second year after birth. Mani and Plunkett, 2010, Mani and Plunkett, 2011 primed 18- and 24-month-olds with images of referents whose labels were either phonologically related or unrelated to the labels of the following target pictures. For example, children saw a picture of a *cat* followed by a picture of a *cup* (phonological related condition) or followed by a picture of a *shoe* (unrelated condition). The results were diametrical for 18- and 24-month-olds. The younger children fixated longer on phonologically primed pictures than on unrelated pictures. Vice versa, the older children fixated longer on unrelated controls than on phonologically primed target images. The authors suggest that the facilitation effect in the 18-month-olds reflects a match between phonological representations and the input. It is only in 24-month-olds that co-activated neighbors with overlapping onsets appear to compete for recognition (McClelland and Elman, 1986).

In sum, results from behavioral paradigms do not unambiguously favor an interpretation of similar word recognition mechanisms in young children and adults. One might argue that different aspects of the complex recognition process are tapped by the different behavioral paradigms. For example, responses in the preferential looking paradigm and phonological priming in referential context might be biased by phonological matching and predictive processing (see Mani and Plunkett, 2010, Mani and Plunkett, 2011). Thus there is a need for measures that are able to disentangle those aspects of speech recognition. In that respect, the recording of ERP responses might provide a promising means for research in the area of early language acquisition.

ERP studies point to a unique neural architecture underlying the processing of familiar words up to 20 months after birth. Across several studies, enhanced ERP negativity for familiar words compared to unfamiliar words has been obtained in infants and young children. This so-called N300-500 effect has been obtained for several instances of familiar words, such as the infants’ own names (Parise et al., 2010), words that parents rated as known to the infants (Mills et al., 1997), or words that had been familiarized during the experiment (Goyet et al., 2010, Junge et al., 2012, Kooijman et al., 2005, Kooijman et al., 2009, Kooijman et al., 2013). In adults, the opposite pattern of ERP amplitudes is usually obtained, namely enhanced negativity for meaningless strings compared to familiar words (N400 pseudoword effect, e.g., Friedrich et al., 2006, Perrin et al., 2005).

ERP indices of semantic integration and phonological expectancy mechanisms are largely comparable between infants and adults. Similar to the semantic N400 effect in adults (for review, see Kutas and Federmeier, 2011), enhanced ERP negativity is found in infants when spoken labels do not match presented pictures (e.g., book–sheep; Friedrich and Friederici, 2004, Parise and Csibra, 2012, Sheehan et al., 2007). N400 modulation in adults is also found in phonological priming (e.g., book–look; Praamstra et al., 1994, Praamstra and Stegemann, 1993, Rugg, 1984), and for mispronunciation in picture-word contexts (e.g., cone–comb; Desroaches et al., 2008). Comparable N400 effects in picture-word contexts, which have been related to phonological matching, were also shown for infants (Duta et al., 2012, Mani et al., 2012, Mills et al., 2004). Together one could conclude from previous ERP studies using different paradigms that phonological matching already is at work in very young infants (phonological N400), while access to familiar words is not adult-like up to 20 months after birth (N300-500 vs. pseudoword N400, see above). This conclusion needs to be validated by means of a single paradigm.

Here we recorded ERPs in auditory word onset priming. In this paradigm, spoken word onset syllables are followed by spoken words. Prime syllables and target onsets either overlap (congruent condition; e.g., in the prime-target pair *ba* – *baby*) or are unrelated (incongruent condition, e.g., *co* – *baby*). ERP effects with different latency and topography have been reliably found when adults (Friedrich et al., 2009, Schild et al., 2012) or children aged 5–8 years (Schild et al., 2011) were tested with this paradigm. Between 100 and 300 ms after target word onset, reduced amplitudes of the N100 complex with central topography have been found for the incongruent condition (but see Schild et al., 2011 for no N100 effect in children). According to the interpretation of the N100 (e.g., Näätänen and Winkler, 1999) this effect has been related to facilitated abstract speech sound processing of congruent targets.

Two effects follow the N100 in word onset priming. Between 300 and 400 ms after target word onset, enhanced negative amplitudes in the congruent condition have been obtained over frontal electrode leads with a left-lateralized maximum at roughly 350 ms (P350 effect). The P350 effect has been related to facilitate access to stored word forms in adults (see also the magnetoencephalographic M350 effect: Pylkkänen and Marantz, 2003; or the P325 effect for written words: Grainger et al., 2006, Holcomb and Grainger, 2006). In parallel to the P350 effect, enhanced negative amplitudes for the incongruent condition have been obtained over centro-posterior electrode leads (central negativity). Its polarity difference and its distribution relate the central negativity to the phonological N400 (Praamstra et al., 1994, Rugg, 1984). In line with that interpretation, we concluded that the central negativity in word onset priming reflects phonological matching between the primes and the targets.

Tracking infants and very young children by means of word onset priming will allow us to arrive at conclusions about word form access on the one hand and predictive phonological processing on the other within a single paradigm. In line with our previous work we suppose enhanced P350 amplitudes to be indicative for word form activation, and enhanced central negativity to be indicative for predictive phonological processing. Our youngest infants were six months old. At this age infants appear to have acquired representations of frequently occurring words (Bergelson and Swingley, 2012, Mandel et al., 1995, Tincoff and Jusczyk, 1999, Tincoff and Jusczyk, 2012). We followed up the first test in 6-month intervals in a quasi-longitudinal design. Our oldest children were 24 months old. Results of behavioral paradigms unambiguously point to adult-like access at this age (Fernald et al., 2001, Mani and Plunkett, 2010, Mani and Plunkett, 2011, Stager and Werker, 1997, Swingley and Aslin, 2000, Swingley and Aslin, 2002). Infants and children's ERP data were compared to that of an adult control group.

## 2 Materials and methods

### 2.1 Participants

Children were recruited from local maternity clinics, newspaper advertisements, or local midwife centers. All participating children had uneventful pre- and perinatal circumstances and had no neurological or developmental problems. All of the children were raised in monolingual German environments. Parents gave their informed consent before the experiment. They received a small present for their children (toy or picture book) for participation. When the child showed any sign of discomfort, a break was inserted during which the parents could comfort the child. The experiment was only continued if both child and parent were happy to do so. The study was approved by the ethical committee of the German Psychological Association (“Ethikkommission der Deutschen Gesellschaft für Psychologie”, 16.04.2010).

In total, 68 children contributed data to one or more age groups, resulting in 36 datasets for the 6-month-olds, 33 datasets for the 12-month-olds, 25 datasets for the 18-month-olds, and 26 datasets for the 24-month-olds (see Table 1). The infants and children were tested repeatedly at 6, 12, 18, and 24 months. In each age group, some children had to be excluded because they fell asleep, or the experiment had to be stopped because they showed signs of discomfort, such as extensive crying before or during the experiment, or they moved extensively or refused to wear the electrode cap. Only the ERPs of participants who contributed at least 15 artifact-free trials per condition were included in the analysis. Note that an exclusion rate of approximately 50% due to artifacts is standard in research with infants and toddlers as young as 6 months (DeBoer et al., 2007). Because of the high drop-out rates, it was not feasible that every child contributed data to all time points.^1^ The mean trial numbers entered in the final analysis are listed in Table 2.

**Table 1:** Demographic data for all four children age groups. Percentage of parental reports of parental left-handedness and developmental speech and language problems in their childhood is given. Note that data for children who contributed to more than one group occur repeatedly in each of the groups where they participated.

Age group	Sex (male/female)	Age in days (mean, range)	Mean gestational age (mean week, range)	Average birth weight (mean weight, SD)	Parental left-handedness	Developmental speech and language problems
6 month	22/14	184 (175–190)	40 (37–42)	3543 g (±410 g)	*n* = 6 (16.7%)	*n* = 3 (8.3%)
12 month	21/12	364 (358–372)	40 (38–42)	3561 g (±441 g)	*n* = 7 (21.2%)	*n* = 2 (6.1%)
18 month	13/12	548 (494–566)	40 (38–42)	3464 g (±381 g)	*n* = 3 (12%)	*n* = 2 (8%)
24 month	17/9	733 (690–733)	40 (38–42)	3444 g (±527 g)	*n* = 3 (11.5%)	*n* = 2 (7.7%)

**Table 2:** Mean number of trials with standard deviation in the grand average per age group, number of interpolated electrodes, and number of excluded children.

Age group	Mean number and standard deviation of trials in grand average (mean; STD for congruent/incongruent)	Number of interpolated electrodes (number of children/number of electrodes)	Additionally tested children who had to be excluded
6 month	24.3; 4.9/23.8; 5.5	9/1, 15/2, 12/3	44
12 month	23.5; 4.3/23.5; 4.5	3/1, 10/2, 16/3	24
18 month	25.6; 4.4/25.6; 3.5	8/1, 1/2, 6/3	13
24 month	27.5; 4.5/27.9; 5.5	5/1, 5/2, 12/3	12
Adults	34.5; 2.6/33.7; 3.4	5/1, 4/2	–

All children successfully passed a TEOAE (Transitory Evoked Otoacoustic Emissions) hearing screening one to three days after birth. The participant characteristics are given in Table 1. Parents were asked to fill out a questionnaire about the medical conditions of their child, parental developmental language impairments, and handedness (Table 1). The parents of the 12- and 18-month-old toddlers completed the ELFRA 1 (Grimm and Doil, 2006), a screening tool for identifying children at risk for later language impairment. The parents of the 24-month-old children completed the ELFRA 2. None of the children were below the threshold for at-risk diagnosis.

Eighteen students of the University of Hamburg (10 females, 8 males; mean age: 25 years, range: 19–33) served as the adult control group. All adult participants were right-handed, as assessed by the Edinburgh Handedness Inventory (Oldfield, 1971), and were native speakers of German without reported hearing or neurological problems. All adult participants gave their informed consent prior to their inclusion in the study. They received course credits or a payment of 8 € for participation.

### 2.2 Stimuli

Forty initially stressed disyllabic German nouns served as stimuli (see Appendix). The words were taken from a German questionnaire assessing early language acquisition milestones (ELFRA). The chosen words are highly frequent in German and are highly likely to occur in everyday parental speech. Prior to the ERP experiment, parents of the 12- and 18-month-olds were asked to rate whether the target words were understood by their children.

We assume that the target words were familiar to the infants because they are frequently used in parental speech. Using the preferential looking technique it has been shown that even 6-month-olds recognize words that are frequently produced by their caregivers, but that it is difficult to validate this assumption on the basis of parental ratings. Parents are likely to underestimate early vocabulary sizes (Bergelson and Swingley, 2012, Houston-Price et al., 2007). Nevertheless, we analyzed parental ratings for the target words based on the ELFRA tests for the 12-month-olds and for the 18-month-olds. In the 12-month-old group, the parents rated only 37% of the target words to be possibly understood by their children (*M* = 15, *SD* = 11). In the 18-month-old group, the parents rated 82% of the words to be possibly understood by their children (*M* = 33, *SD* = 6). In an additional analysis we compared the results for words the parents rated as known or unknown by their children (see Section 3).

Stimuli were spoken by a female and a male professional native speaker of German. The speakers were unaware of the specific aims of the study. To increase the children's attention to the stimuli, words were spoken in infant-directed speech. Using Adobe Audition^®^ software, primes and targets were edited from digitized speech (sampling rate 44.1 kHz). The primes were first syllable fragments taken from the words. The targets were the complete words. The primes were taken from the utterances of the female speaker, and the targets were taken from the utterances of the male speaker. The volume was equalized for all stimuli.

### 2.3 Design and procedure

Throughout the experiment, the children sat on their parent's laps, and the adult control group sat comfortably on a chair. To ensure a similar recording situation between children and adults, the adult control group was not instructed to pay specific attention to the words but just to sit silent, listen and watch a movie. The EEG recording took place in an electrically shielded and sound-attenuated chamber. To avoid movement artifacts, we presented a silent movie that consists of short scenes (e.g., two girls laughing, a dolphin swimming), but without telling a story, as is appropriate for capturing the attention of infants (Baby Einstein^®^, Walt Disney Studios, 2007). The movie appeared on a computer screen in front of the participants. Note that this is common practice in studies with infants and children (e.g., Grossmann et al., 2010, Wartenburger et al., 2007). The movie was started during the preparation of the EEG at different time points for each participant. This procedure ensured that the presentation of the auditory stimuli never started with the same events in the movie. The auditory stimuli were presented via two loudspeakers placed at the left and right sides of the screen (sound level of approximately 65 dB), at a distance of approximately 1 m from the participants. Stimulus presentation was controlled by Presentation^®^ software (version 14.9, Neurobehavioral Systems).

**Fig. 1:**
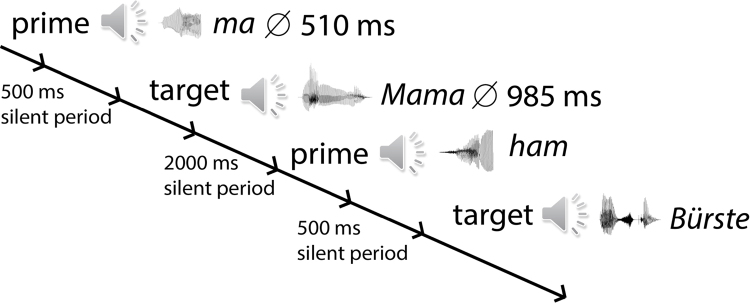
Trial structure. In each trial, after the offset of the prime (mean duration 510 ms, range = 277–818 ms), a 500 ms silent period followed. Then, the target (mean duration 985 ms, range = 743–1150 ms) was presented. The next trial started after a 2.0-s inter-trial interval. The total duration of the experiment was approximately 6 min.

The primes and target words were combined according to their overlap. The prime was the onset of the target word in the congruent condition, such as *ma* – *Mama*. There was no segmental overlap between prime and target in the incongruent condition, such as *so* – *Mama*; see Fig. 1 for the trial structure. The experiment consisted of 80 trials. All 40 primes and all 40 targets were presented twice: once in the congruent condition and once in the incongruent condition. The trials were presented in two blocks. In each block, each target and each prime occurred only once. Twenty trials in each block were congruent, and 20 were incongruent. Within each block, the trials were presented in a total randomized order. Half the experiments started with one of the two blocks, the other half of the experiments started with the other block. That is, each target was equally likely to occur first in the congruent condition or in the incongruent condition.

### 2.4 Electrophysiological recording and data analysis

**Fig. 2:**
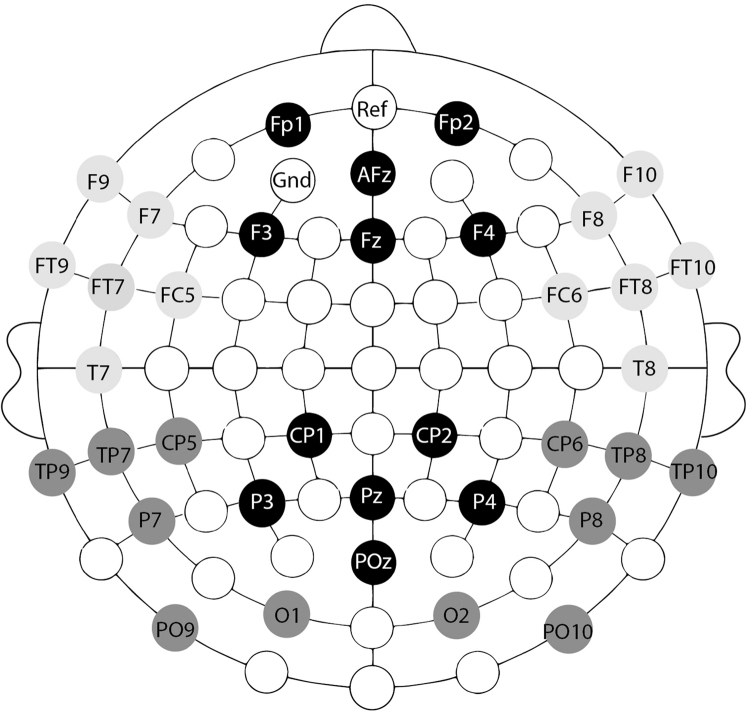
Regions of interest. Mean amplitudes for the time windows were calculated for two central and four lateral regions of interest (ROI). Each ROI consists of six electrodes (left anterior: F9, F7, FT9, FT7, FC5, T7; right anterior: F10, F8, FT10, FT8, FC6, T8; both marked in bright gray; posterior left: TP9, TP7, CP5, P7, PO9, O1; posterior right: TP10, TP8, CP6, P8, PO10, O2; both marked in dark gray; midline anterior: FP1, AFz, FP2, F3, Fz, F4; midline posterior: CP1, CP2, P3, Pz, P4, POz; both marked in black).

The continuous EEG (500 Hz/22 bit sampling rate, band pass 0.01–100 Hz) was recorded for 45 active Ag/AgCl electrodes (Brain Products) mounted in an elastic cap (Electro Cap International Inc.) according to the international 10–20 system. See Fig. 2 for the electrode positions. The FPz was used as an online reference; the ground electrode was positioned at AF3, see Fig. 2.

Offline analysis was performed using BESA-Research software (^®^MEGIS Software GmbH; Version 5.3). After filtering the data with a high-pass filter of 0.5 Hz, the continuous EEG for very noisy channels was estimated by a linear interpolation of adjacent electrodes (Picton et al., 2000). No low-pass filter was applied offline. Table 2 shows the number of interpolated electrodes per group. The interpolated EEG was re-referenced to an average reference. Artifacts were rejected manually according to a visual inspection of each individual continuous EEG. Trials with artifacts were removed from the analysis. For the adult data, we used the same online and offline filters, and the same interpolation procedures as for children. In addition, we applied automatic eye movement correction to the adult data (Multiple source eye correction by Berg and Scherg, 1994, implemented in BESA). Blinks and horizontal eye movements were corrected from the continuous EEG (re-referenced to an average reference, including interpolated channels). This procedure was identical to our former word onset priming studies with adults (e.g., Friedrich et al., 2009, Schild et al., 2012).

The target word ERPs were computed starting from the beginning of the auditory presentation of the target word up to 1200 ms using a 200 ms pre-stimulus baseline for each condition. A repeated measures analyses of variance (ANOVA) with the within factor *Condition* (congruent vs. incongruent) and the between factor *Age group* (6 months vs. 12 months vs. 18 months vs. 24 months vs. adult control group) was performed to determine whether the number of excluded segments differed across conditions and age groups. There was a significant effect for the factor *Age group, F*(4,133) = 24.43, *p* < .001, pointing to differences in the total number of trials between the groups. Most trials were obtained from adults. The least number of trials was obtained from 12-month-olds (see Table 2). Neither the factor *Condition, F*(1,133) = 0.13, *p* = .72, nor the interaction of the factors *Condition* and *Age group, F*(1,133) = 0.30, *p* = .88, reached significance.

To analyze the adult data, we relied on our previous studies with adults. Hence, we calculated six ROIs (regions of interest; see Friedrich et al., 2009, Schild et al., 2012) containing six electrodes each (see Fig. 2); and we applied two predefined time windows, ranging from 100 to 300 ms and from 300 to 400 ms after target word onset (see Friedrich et al., 2009).

To analyze the infant and children data, we first calculated repeated measurement ANOVAs with the within-subject factors *Condition* (congruent vs. incongruent), *Hemisphere* (left vs. middle vs. right ROIs), and *Region* (anterior vs. posterior ROIs), and with the between-subject factor *Age group* (6 months vs. 12 months vs. 18 months vs. 24 months). We are aware of the fact that a repeated measure ANOVA for our design is problematic. Measurements at the different time points are partially obtained from the same participants. This confound is related to our attempt to run a longitudinal design; and to procedural advances in realizing repeated testing (see Section 2.3). Unfortunately, sample sizes per cell were too low to run mixed models. Therefore, we opted to handle *Age Group* as a between-subject factor to determine effects that are similar over all children groups and to estimate time windows of interest for separate ANOVAs per group.

We started with 50 ms consecutive time window analyses (from 0 to 1200 ms) to determine the onsets and offsets of significant differences between conditions and interaction with the factor *Age Group* (see Table 3). This analysis resulted in two larger time windows, for which no interaction with the factor *Age Group* was evident: 150–300 ms and 550–650 ms; and two larger time windows, for which an interaction of *Age Group* and *Condition* was evident respectively: 350–500 ms and 650–1050 ms. Follow-up ANOVAs for the single age groups were conducted when an interaction of *Condition* with *Age Group* was significant.

**Table 3:**
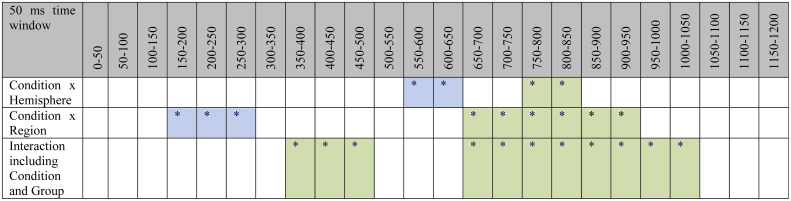
50 ms time window analyses of the ERP data of the four children groups and larger time windows that were further analyzed.

(*) *p* ≤ 0.1.

^*^p ≤ 0.05.

^**^p ≤ 0.01.

^***^p ≤ 0.01.

In all analyses, the Greenhouse–Geisser Epsilon (*ɛ*) correction was applied to effects including the three-level factor *Hemisphere*, and corrected *p* values were reported. In the case of significant interactions, *t*-tests were computed to evaluate differences among the conditions. Only the main effects of the factor *Condition*, significant interactions with *Condition*, and significant post hoc comparisons are reported.

## 3 Results

### 3.1 Adults

**Fig. 3:**
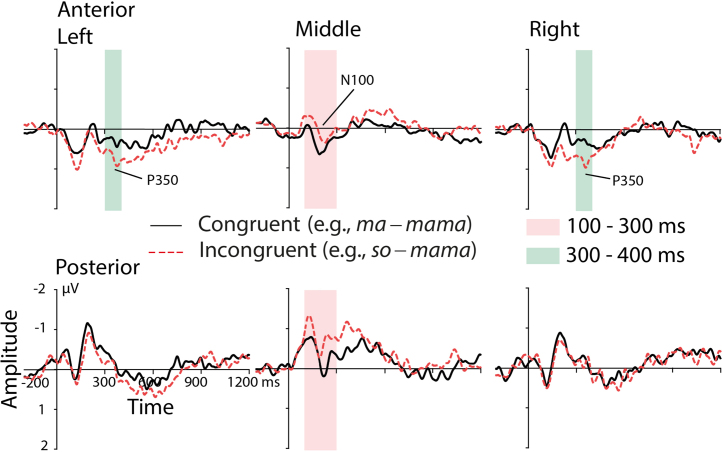
ERPs for the congruent condition (solid black lines) and for the incongruent condition (dashed red lines) obtained from the adult group over all six ROIs. Significant differences between the congruent condition and the incongruent condition are highlighted (i) in light pink for the N1 and (ii) in light green for the P350 effect. For illustration purposes, the ERPs were filtered with a 20 Hz low-pass filter. (For interpretation of the references to color in text, the reader is referred to the web version of this article.)

Fig. 3 illustrates the ERPs for the congruent and incongruent condition for all six ROIs recorded from the adults. According to our previous research with auditory word onset priming (Friedrich et al., 2009), we analyzed two corresponding time windows. ERPs in the early time window ranging from 100 to 300 ms reflect the N100 complex. ERPs in the later time window from 300 to 400 ms rather relate to the P350 effect (enhanced negativity for the congruous condition and frontal distribution of the effect) than to the central negativity.

#### 3.1.1 100–300 ms

The ANOVA revealed a significant interaction of the factors *Condition* and *Hemisphere, F*(2,34) = 5.23, *p* = .01, *ɛ* = 0.89. Post hoc *t*-tests pointed to significant differences over midline electrode sites, *t*(17) = 3.85, *p* < .01. The ERPs for the incongruent condition were more negative (*M* = −0.39 μV, *SD* = 0.43) than the ERPs for the congruent condition (*M* = −0.03 μV, *SD* = 0.31) (see Fig. 3). This result corresponds in latency and polarity to the formerly described N100 effect (Friedrich et al., 2009, Schild et al., 2012).

#### 3.1.2 300–400 ms

The ANOVA yielded a significant interaction of the factors *Condition* and *Region, F*(1,17) = 6.39, *p* = 02. The *t*-tests showed that the effect was significant over the anterior electrode sites, *t*(17) = −2.96, *p* < .01. The ERPs for the incongruent condition were more positive (*M* = 0.55 μV, *SD* = 0.37) than the ERPs for the congruent condition (*M* = 0.19 μV, *SD* = 0.52). The anterior distribution and the polarity of amplitudes for the congruent and incongruent conditions are comparable to the formerly described P350 effect (e.g., Friedrich et al., 2009). However, in the present study we did not find hints for a left-lateralized topography of this effect.

### 3.2 Infants and young children

**Fig. 4:**
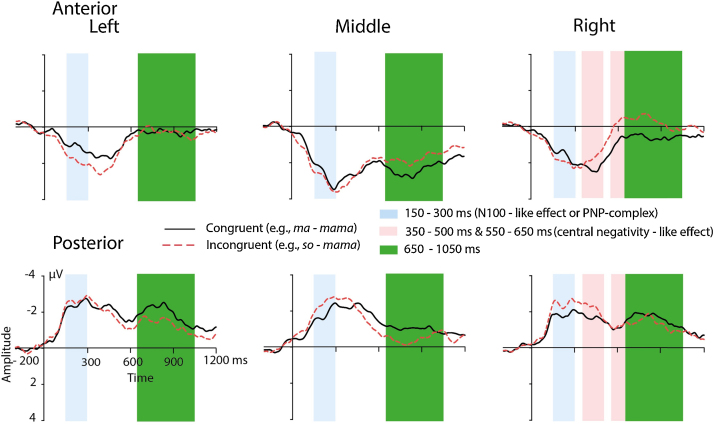
ERPs (over all six ROIs) for the congruent condition (solid black lines) and for the incongruent condition (dashed red lines) averaged over all four children groups. Significant differences between the congruent and the incongruent condition that were consistent across all children groups are highlighted. For illustration purposes, the ERPs were filtered with a 20 Hz low-pass filter. (For interpretation of the references to color in text, the reader is referred to the web version of this article.)

**Fig. 5:**
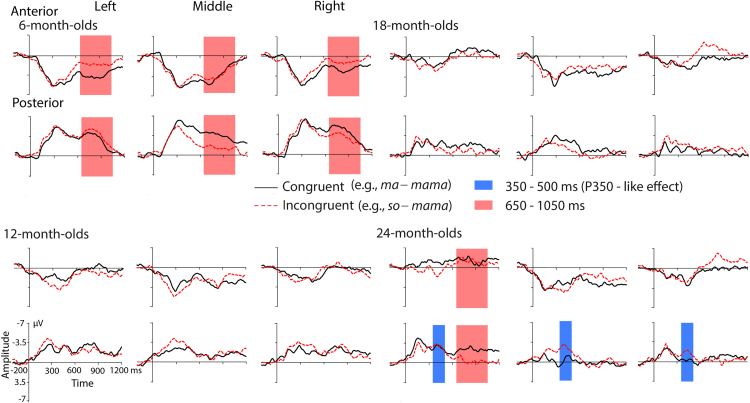
ERPs (over all six ROIs) for the congruent condition (solid black lines) and for the incongruent condition (dashed red lines) separately shown for the four children groups. Unique differences between the congruent condition and the incongruent condition for the 6-month-olds and the 24-month-olds are highlighted. For illustration purposes, the ERPs were filtered with a 20 Hz low-pass filter. (For interpretation of the references to color in text, the reader is referred to the web version of this article.)

Fig. 4 displays averaged ERPs over all four children age groups. Fig. 5 illustrates ERPs of the single groups. We first conducted 50-ms time window combined analyses of all children groups (see Table 3). Time windows with two or more consecutive significant 50 ms intervals were merged into larger time windows and further analyzed. Based on consecutive time windows showing condition effects, four larger time windows were analyzed in more detail. A first time window, ranging from 150 to 300 ms, captures the first consecutive condition effects in the 50 ms time-step analyses. Furthermore, the ERPs in this early time window approximately correspond to the adult N100 and its immature instance, the infant PNP complex (Duta et al., 2012, Wunderlich and Cone-Wesson, 2006). A second time window with no interaction with the factor *Age Group* lasted from 550 to 650 ms. From 350 to 500 ms and from 650 to 1050 ms, an interaction of *Condition* and *Age Group* was seen. Consecutive step-down ANOVAs in the single groups were conducted for both time windows.

#### 3.2.1 150–300 ms

The combined ANOVA revealed a significant interaction of the factors *Condition* and *Region, F*(1,116) = 10.23, *p* < .01. The post hoc *t*-tests pointed to significant differences between both conditions over anterior and posterior electrode sites. Over anterior electrodes, amplitudes for the incongruent condition were more positive (*M* = 2.85 μV, *SD* = 2.22) than those for the congruent condition (*M* = 2.20 μV, *SD* = 2.22), *t*(119) = −3.58, *p* = .001. Over posterior electrodes, amplitudes for the incongruent condition were more negative (*M* = −3.16 μV, *SD* = 2.51) than amplitudes for the congruent condition (*M* = −2.50 μV, *SD* = 2.29), *t*(119) = 3.07, *p* < .01. No significant interaction of the factors *Condition* and *Group* was seen in this time window, which approximately corresponds to that of the time window of the adult N100.

#### 3.2.2 350–500 ms

The ANOVA in this time window revealed a significant interaction of the factors *Condition* and *Hemisphere, F*(2,232) = 3.27, *p* ≤ .05, *ɛ* = 0.89. Post hoc comparisons pointed to significant differences between both conditions over the right hemisphere, *t*(119) = 1.99, *p* ≤ .05. Averaged across all infant groups, ERP amplitudes were more negative to the incongruent condition (*M* = −0.34 μV, *SD* = 2.52) than to the congruent condition (*M* = 0.24 μV, *SD* = 2.60). There was no interaction of the factors *Condition, Hemisphere* and *Group, F* < 1.

In addition to the overall right-lateralized ERP effect that was evident across all groups (see above), a significant interaction of *Group, Condition* and *Region* pointed to different effects for infant and young children at different ages, *F*(3,116) = 3.58, *p* = .02. Follow up-ANOVAs including the factors *Region* and *Condition* were calculated for each single group. Results are summarized in Table 4. Only in the 24-month-olds, there was a significant interaction between the factors *Region* and *Condition*. The post hoc *t*-test for posterior electrodes revealed more negative amplitudes for the incongruent condition (*M* = −2.37 μV, *SD* = 2.79) than for the congruent condition (*M* = −1.18 μV, *SD* = 1.89), *t*(25) = 2.39, *p* = .02. There was no difference between conditions for the anterior electrodes.

**Table 4:** Results of follow-up ANOVAs in the time window from 350 to 500 ms over all four children groups.

Age group	*df*	*F*	Age group	*df*	*F*
**6-month**			**18-month**		
*Condition*	1.35	0.08	*Condition*	1.24	0.06
*Condition* × *Region*	1.35	3.79	*Condition* × *Region*	1.24	0.58
**12-month**			**24-month**		
*Condition*	1.32	0.01	*Condition*	1.25	1.07
*Condition* × *Region*	1.32	2.46	*Condition* × *Region*	1.25	5.06^*^

^*^p ≤ 0.05.

^**^p ≤ 0.01.

^***^p ≤ 0.01;

#### 3.2.3 550–650 ms

The examination of this time window revealed a significant interaction of *Hemisphere* and *Condition, F*(2,232) = 3.3, *p* = .04, *ɛ* = .99. Post hoc *t*-tests indicated a significant difference between both conditions over the right hemisphere, *t*(119) = 2.14, *p* = .03. Amplitudes were more negative for the incongruent condition (*M* = −0.85 μV, *SD* = 2.46) than for the congruent condition (*M* = −0.27 μV, *SD* = 2.29). This effect seems to be an extension of the overall effect seen in the preceding time window ranging from 350 to 500 ms (see also Fig. 4). Together results in both time windows reflect sustained right-lateralized negativity for incongruent targets.

#### 3.2.4 650–1050 ms

In this time window, a significant interaction of the factors *Region* and *Condition* was observed, *F*(1,116) = 6.56, *p* ≤ .01. Over anterior electrode sites, amplitudes for the incongruent condition were more negative (*M* = .91 μV, *SD* = 1.99) than amplitudes for the congruent condition (*M* = 1.48 μV, *SD* = 2.32), *t*(119) = 2.59, *p* ≤ .01. Over posterior electrode sites, amplitudes were more positive for the incongruent condition (*M* = −1.47 μV, *SD* = 2.18) than for the congruent condition (*M* = −2.01 μV, *SD* = 2.32), *t*(119) = −2.51, *p* ≤ .01.

In addition, a significant interaction of the factors *Hemisphere, Condition* and *Group* was seen in this time window, *F*(6,232) = 3.57, *p* = .002, *ɛ* = .99. Follow-up ANOVAs including the factors *Hemisphere* and *Condition* were calculated for the single groups of infants and children. Results are summarized in Table 5. The group of the 6-month-olds showed a significant main effect of *Condition*, with more negative ERP amplitudes to the incongruent condition (*M* = −0.35 μV, *SD* = 0.50) than to the congruent condition (*M* = −0.22 μV, *SD* = 0.56). Only for the group of the 24-month-olds, there was a significant interaction of the factors *Condition* and *Hemisphere*. The post hoc *t*-test for the left hemisphere revealed more positive amplitudes for the incongruent condition (*M* = −.65 μV, *SD* = 1.79) than for the congruent condition (*M* = −1.73 μV, *SD* = 1.44), *t*(25) = −2.85, *p* ≤ .001. There was no significant difference for the right-hemispheric electrodes.

**Table 5:** Results of follow-up ANOVAs in the time window from 650 to 1050 ms over all four children groups.

Age group	*df*	*F*	Age group	*df*	*F*
**6-month**			**18-month**		
*Condition*	1.35	11.64^**^	*Condition*	1.24	0.31
*Condition* × *Hemisphere*	2.70	1.69	*Condition* × *Hemisphere*	2.48	2.75
**12-month**			**24-month**		
*Condition*	1.32	0.31	*Condition*	1.25	1.44
*Condition* × *Hemisphere*	2.64	0.79	*Condition* × *Hemisphere*	2.50	8.46^***^

^*^p ≤ 0.05.

^**^p ≤ 0.01.

^***^p ≤ 0.01.

### 3.3 Familiarity effects (according to parental ratings) for 12-month-olds

For the 12-month-olds, we conducted an analysis for words that were rated as understood vs. not-understood by the parents. Data of 28 children entered this analysis. Five children had to be excluded: For two of them, the parents rated all words as known. For two of them, no trials remained in the unknown condition after artifact correction. For one child, the experimental protocol allowing identifying the single target words was not available due to technical problems during the recording. The mean trial numbers between words rated as known (*M* = 17.8, *SD* = 11.1) and words rated as unknown (*M* = 32.6, *SD* = 14.2) differed significantly, *t*(27) = −3.3, *p*< .01. Mean ERP amplitudes were analyzed by means of a repeated measure ANOVA with the factors *Hemisphere* (left vs. middle vs. right), *Region* (anterior vs. posterior), *Understanding* (understood vs. misunderstood) and *Condition* (congruent vs. incongruent). 50 ms analyses as described above were conducted. In no time window, a significant effect of *Condition, Understanding* or interactions with one of these factors were obtained. That is, the pattern of ERP results does not appear to differ between words that parents rated as known or unknown to their infants. In the group of the 18-month-olds, not enough trials rated as unknown remained to make comparisons possible.

## 4 Discussion

By means of ERPs recorded in auditory word onset priming, the present study explored access to phonological representations and predictive phonological processing in infants and toddlers aged 6, 12, 18, and 24 months, and in adults. The participants heard the onsets of words that are frequently used in German parental speech. The spoken word onsets were either followed by the same complete target word (congruent condition, e.g., *ma* – *Mama*) or by a target word with a different onset (incongruent condition, e.g., *so* – *Mama*). To make the paradigm suitable for children this young, we had to present our primes and targets in a passive listening paradigm. Because former word onset priming studies with adults and children included a lexical decision task in which participants had to decide whether the target is a word or not (Friedrich et al., 2009, Schild et al., 2011), we first evaluate whether a passive listening situation in an adult control group elicits the ERP deflections formerly reported.

The present ERPs for adults largely replicate previously observed N100 and P350 effects in unimodal auditory word onset priming that were obtained when adult participants were engaged in a lexical decision task. We interpret these effects in accordance with previous work (e.g., Friedrich et al., 2009, Schild et al., 2011). The midline N100 effect between 100 and 300 ms suggests that congruent word onset primes modulate abstract speech sound processing for their subsequent targets. The bilateral anterior P350 effect between 300 and 400 ms suggests that access to phonological representations is modulated by the prime word onsets. However, we did not replicate the formerly observed left lateralization of either effect. This might be related to the passive listening situation applied in the present study. Others have already reported that the degree of lateralization of ERP effects in language processing is modulated by the type of task in which the participants are involved (Rowan et al., 2004, Rugg, 1984, Spironelli and Angrilli, 2006). For example, right-lateralization to normal vs. flattened speech was only found when participants were engaged in an active task, but not when they were engaged in passive listening (Plante et al., 2002). Together, the N100 and P350 effect suggest that automated aspects of the complex spoken word recognition stream, namely abstract speech sound processing and access to stored word forms, can be elicited in a passive paradigm. Further studies with word onset priming should investigate whether lateralization of both effects is due to processing associated with the lexical decision task.

The central negativity formerly which was obtained in unimodal fragment priming studies when children and adults were engaged in a lexical decision task (Friedrich et al., 2009, Schild et al., 2011, Schild et al., 2012) was not replicated in the present study with adults. One might conclude that the central negativity effect in adults reflects processing in word onset priming, which is not obligatorily elicited in spoken word recognition, but is related to the lexical decision task requiring participants to indicate as rapidly and as accurately as possible whether or not the target is a word. The lexical decision task might force participants to initiate fast phonological matching and/or to rely on predictive processing in order to speed up their responses. Given the present failure to show a central negativity without a task, it appears that adults do not obligatorily exploit the prime to predict the upcoming target in a passive listening situation. Further research with adults has to evaluate this interpretation.

The enhanced statistical power of the omnibus analysis including 120 datasets pointed to generalizable effects in all children groups starting between 150 and 300 ms after target word onset. In the early time window, which roughly corresponds to the adult N100 time window, ERP morphology is characterized by successive positive and negative peaks (PNP complex). Formerly, the PNP complex has been related to a premature instance of the adult N100 (Wunderlich and Cone-Wesson, 2006, Duta et al., 2012). That is, we find an N100-like effect with young children aged 6–24 month in the present study. This finding somewhat contrasts with a previous study with preschool children and young pupils, in which an N100 effect was visible in the data but did not reach significance between conditions (Schild et al., 2011). The lower number of participants (51 children) in the former study might be a possible explanation by indicating that statistical power is a critical aspect in ERP language acquisition research. The PNP priming effect in the present study, in which primes and targets were spoken by different speakers, suggests that the underlying mechanisms must operate on abstract speech sound representations. The finding that even 6-month-olds can normalize across acoustic variations such as those related to different voices is in line with behavioral research (Jusczyk et al., 1992, Kuhl, 1983).

A second consistent ERP effect in the combined analysis of all children groups was an enhanced negativity for incongruently primed words over the right hemisphere between 350 and 650 ms. Because the effect occurs after the offset of the first syllable of the target, we suppose that it reflects more than a pure one-to-one syllable matching process or repetition priming. In more general, polarity, latency and distribution relate this effect to the phonological N400 effect that has been obtained in picture-word contexts in adults and children (Duta et al., 2012, Mani et al., 2012, Mills et al., 2004). With respect to auditory word onset priming, polarity, latency and distribution relate this effect to the central negativity, which has previously also been related to the phonological N400 in adults and children (Friedrich et al., 2009, Schild et al., 2011, Schild et al., 2012). According to the interpretation of the central negativity in adults, the present data suggest that infants as young as 6 months old appear to match phonological predictions established by the primes to predict highly frequent phonological word forms. Following this line of interpretation, our results suggest commonalities for predictive phonological processing in adults (at least when a psycholinguistic task is involved) and young children from the age of 6 to 24 months.

In the overall group analysis including all children, we did not obtain evidence for an ERP deflection comparable to the P350. One might conclude that young children do not use word onsets for activating phonological representations in the way adults do, and this might be generalized to the assumption that adult-like access to word form representations is not established in the first two years after birth. Only the 24-month-olds showed a hint for a left-lateralized P350-like deflection. This conclusion also is in line with previous behavioral and ERP research. Fixation data in phonological priming contexts revealed that competition effects, which characterize phonological access in adults, develop only slowly at the end of the second year of life (Mani and Plunkett, 2010, Mani and Plunkett, 2011). ERPs show a unique effect for familiar words up to 20 months after birth (Goyet et al., 2010, Junge et al., 2012, Kooijman et al., 2005, Kooijman et al., 2009, Kooijman et al., 2013, Mills et al., 1997), which substantially differs from the adult pseudoword N400 effect (e.g., Friedrich et al., 2006, Perrin et al., 2005).

In a time window ranging from 650 to 1050 ms after target word onset, we observed very late ERP effects in all children groups. This finding has no parallel in the adult data. However, long-lasting ERP effects are often reported in infant studies (e.g., Friedrich and Friederici, 2004, Friedrich, 2005). In the present study, they might be related to extended processing of the phonological match and/or mismatch between prime and target.

## 5 Conclusion

Together the present results characterize word onset priming as a promising means for investigating several aspects of adult word recognition in infants within a single paradigm. It appears that infants as young as 6 months old operate on abstract phonological representations of the prime syllables and use those them to make predictions about the upcoming targets. However, infants and young children did not show a robust index of adult-like lexical access to stored phonological forms. Therewith, ERPs recorded in word onset priming might allow characterizing aspects of word recognition and the format of phonological representations in infancy in more detail than behavioral measures and previously used ERP paradigms allowed.

## Appendix A German words that were presented in both the congruent and incongruent conditions

Ap-fel [apple]

Au-to [car]

Ba-by [baby]

Bir-ne [pear]

Blu-me [flower]

Bril-le [glasses]

Dok-tor [doctor]

Do-se [can]

En-te [duck]

Es-sen [food]

Fen-ster [window]

Fla-sche [bottle]

Ga-bel [fork]

Haa-re [hair]

Ha-se [rabbit]

Ho-se [trousers]

Kä-se [cheese]

Kat-ze [cat]

Kü-che [kitchen]

Ku-chen [cake]

Lam-pe [lamp]

Lip-pen [lips]

Löf-fel [spoon]

Mäd-chen [girl]

Ma-ma [mommy]

Mes-ser [knife]

Müt-ze [hat]

Na-se [nose]

Pa-pa [daddy]

Pup-pe [doll]

Rut-sche [slide]

Sche-re [scissors]

Schlüs-sel [key]

Soc-ken [sock]

So-fa [couch]

Sup-pe [soup]

Tas-se [cup]

Trak-tor [tractor]

Trep-pe [stairs]

Vo-gel [bird]

## Footnotes

1 Thirty-two children contributed data to only one grand average, 21 children to two grand averages, 13 children to three, and only two to all four grand averages. Sixteen children were tested who did not contribute data to any of the groups.
